# Is treatment of fatty liver effective on reducing carotid intima media thickness; a cohort study

**Published:** 2018

**Authors:** Saeideh Ghaffarifar, Manouchehr Khoshbaten, Fattaneh Dordaei, Maryam Zareh Nahandi, Reza Javad-Rashid, Tahereh Shahnazi

**Affiliations:** 1 *Medical Education Research Center, Health Management and Safety Promotion Research Institute, Tabriz University of Medical Sciences, Tabriz, Iran*; 2 *Gastroenterologist, Drug Applied Research Center (DARC), Tabriz University of Medical Sciences, Tabriz, Iran*; 3 *Internist, Drug Applied Research Center (DARC), Tabriz University of Medical Sciences, Tabriz, Iran *; 4 *Drug Applied Research Center (DARC), Tabriz University of Medical Sciences, Tabriz, Iran*; 5 *Radiologist, Drug Applied Research Center (DARC), Tabriz University of Medical Sciences, Tabriz, Iran*; 6 *Drug Applied Research Center (DARC), Tabriz University of Medical Sciences, Tabriz, Iran*

**Keywords:** Carotid, Intima Media thickness, Color Doppler Sonography, Fatty Liver

## Abstract

**Aim:**

This study was intended to explore the effect of various drugs used to treat fatty liver on intimal-media thickness in patients with NAFLD.

**Background:**

Nonalcoholic fatty liver disease (NAFLD) is an indicator of a broad spectrum of pathologic disorders, which is characterized with macro vesicular steatosis in the absent of alcohol use. It has a wide range of laboratory, clinical and pathological presentations such as simple steatosis to the diseases like non-alcoholic steatohepatitis, fibrosis, and cirrhosis and hepatocellular cancer.

**Methods:**

In this cross - sectional study, as a part of a 10-year cohort study (from 2007-2017) at Tabriz University of Medical Sciences, a group of 100 patients with NAFLD were studied. They were examined by color doppler sonography of the carotid arteries to detect any carotid intima- media thickness, before and one year after treatment with various drugs. The effect of treatment on right and left carotid intima- media thickness (IMT) was examined by using SPSS. V21.

**Results:**

Over all, 36 (36%) patients were male and 64 (64%) were female. The mean age of the patients was a 43.5±10.3 year, ranging from 16 to 64. The decrease in patients' intima- media thickness in both right and left carotids was statistically significant (P<0.0001).

**Conclusion:**

Treatment of patients with nonalcoholic fatty liver has a significant role in reduction of their carotid intima -media thickness and consequently in reducing cerebrovascular events such as stroke.

## Introduction

 The prevalence of non-alcoholic fatty liver disease (NAFLD) is different in the world; however, epidemiological studies in western countries indicate that NAFLD is seen in 30-20% of the people and the findings in the liver biopsy of patients with non-alcoholic liver disease are similar (1). In the United States, it is believed that the prevalence of non-alcoholic steatohepatitis (NASH) is 3% and NASH-induced fibrosis is seen in 40% of obese patients (2). Approximately, 20% of patients with NASH can progress to cirrhosis over a decade, while simple steatosis or fatty liver is itself a benign condition. With increasing prevalence of obesity in many western countries, it is not surprising that NAFLD, which is associated with obesity, is a common health problem. The best current estimates reported for the prevalence of NAFLD and NASH are 20% and 2 - 3% respectively (3).

NAFLD includes a wide range of laboratory, clinical and pathological presentations, which vary from simple steatosis to diseases like non-alcoholic steatohepatitis, fibrosis, cirrhosis and hepatocellular cancer (4). Association of NAFLD with obesity and insulin resistance is common, too. Atherosclerosis in obese people with NAFLD is more common and faster than the obese people with no NAFLD progresses. The first symptoms of atherosclerosis are the fatty streaks. In the presence of hyperlipidemia, smoking, hypertension, obesity and diabetes mellitus, progression of the symptoms becomes faster (5). The rapid diagnosis of the lesions and the elimination of the risk factors can lead to the slow progression and even regression of the lesions. Recent advances in imaging and using ultrasound technique have made the early diagnosis of vascular changes possible. The early changes include thickening of the vessel wall, arterial stiffness and arterial vasodilator function disorder (6). 

Various studies showed that the measurement of intimal- media artery thickness was a superior marker for diagnosing subclinical atherosclerosis. Moreover, recent studies have shown that intima -media artery thickness (IMT) in patients with NAFLD was higher than the normal ones (5).

Some studies in the world have been conducted about the effect of treatment of fatty liver disease on reducing carotid intima media thickness. Those studies have not been integrated and each of them has examined the treatment effect of an individual drug on the thickness of intima in just a specific perspective. 

Thus, considering the points that few studies have been conducted for the treatment of the patients with NAFLD and no definitive treatment for NAFLD has been specified yet and in order to test the hypothesis that treatment of non-alcoholic fatty liver disease can decrease carotid artery intima-media thickness as a risk factor for atherosclerosis in patient with NAFLD, the researchers decided to examine the effect of any successful drug therapy of fatty liver in patients with NAFLD on intima-media thickness regardless of the type of medication. 

## Methods

In this cross - sectional study, as a part of a 10-year cohort study (from 2007-2017) at Tabriz University of Medical Sciences, a group of 100 patients with NAFLD were studied. Patients were visited in Gastroenterology Clinics of Imam Reza teaching hospital or Sheikh Alraeis medical center. 

Proportion estimation formula was used to determine the sample size. Considering α = 0.05, d = 0.14, p = 0.5 and power of 80%, 98 samples were estimated. In order to increase the validity of the research conclusion and considering any probable sample loss, 100 patients were studied.

Patients with NAFLD, who met criteria for metabolic and homogenized syndrome, were recruited to participate in the study. Other inclusion criteria were desire to participate in the study and having fatty liver diagnosed with sonography. Exclusion criteria were consuming more than 20 g of alcohol daily; having drug induced hepatic steatosis; having positive markers of viral or autoimmune diseases or having laboratory evidence confirming Wilson disease or hemochromatosis.

In this study, vitamin E was given daily at a dose of 400 mg. This dose is the minimum prescriptive dose in studies. In various studies, it has been prescribed for up to 1200 mg too. 

The use of Vitamin E may cause allergic reactions and symptoms such as itching in patients. Symptoms of intolerance and common side effects such as nausea, vomiting, nervous and digestive problems in the dose of 400 mg are usually not seen. Vitamin E was well tolerated by all our patients and no cases of non-consumption due to intolerance were reported among our patients and such symptoms were not seen in our patients.

Until the time of this study, there was no standard choice for the treatment of fatty liver in the existing guidelines and researchers had suggested different ways to treat fatty liver over the past years, which often their effectiveness had not been verified by later studies. So, in this study, using the data from a 10-year cohort study, every time a new drug was introduced for treatment of fatty liver, that new drug, with its recommended dose, was being prescribed to patients.

First, all participants were examined by color doppler sonography of the carotid arteries to detect any carotid intima- media thickness. 100 patients, who had carotid intima- media thickness, were treated by various drugs such as Gemfibrozil, UDCA, vitamin E, vitamin C and metformin for one year.

In fact, various drugs, that were the best drugs for treatment at their time, were prescribed to patients at different times of this study and the effect of any prescribed drugs on the thickness of intima is reported, regardless of the type of medication. 

Then, all patients, who had received treatment, were followed for at least 12 months. At the end of the treatment, a skilled sonographer did liver sonography for all patients and patients’ intima- media thickness was measured with color doppler sonography, again. 

The data from the patients, who had completed their treatment course and their treatment was successful, were analyzed, using SPSS version 21.and P-value less than 0.05 was considered statistically significant. T-test was used to compare quantitative variables and Qui-square test for qualitative variables. Fisher's test was used when necessary. 

## Results

All 100 patients successfully completed all phases of the study. Overall, 36 patients (36%) were male and 64 (64%) were female. The mean age of the patients was 43.5±10.3 years, ranging from 16 to 64.

Only 4 patients (4%) had positive previous medical history. One participant had used estrogen. One and two other patients had used losartan and atenolol, respectively. Among the underlying diseases, history of hypertension (HTN) was negative in 88 patients (88%).16 patients (16%) were suffering from diabetes and 21 (21%) had hyperlipidemia (HLP).

Some opatients' demographic characteristics, such as their weight, height, body mass index (BMI), waist circumference and their blood pressure before intervention are shown in Table 1.

Patients’ liver function tests (ALT, AST and ALP) and their comparison before and after treatment are revealed in Table 2. Patients’ carotid intima media thickness in both right and left sides and their comparison before and after treatment are shown in Table 3.

**Table 1:** Demographic characteristics of the patients (n=100) with NAFLD before intervention*.

Variable	Mean±SD	Range
Height(cm)	162.9±9.9	(145-185)
Weight(kg)	79.7±12.3	(56-115)
BMI( kg/m2)	29.9±4	(23.2-42.7)
Waist Circumference(cm)	102.1±8.2	(83-130)
FBS	95.8±20.2	(64-190)
TG	163.9±10.5	(37-727)
Total Chol	188.7±46.9	(30-303)
LDL	108.1±31.3	(49-184)
HDL	45±13.9	(29-51)

* Patients, who had carotid intima- media thickness in their color doppler sonography, received treatment.

**Table 2:** Comparison of patients*’ liver enzymes before and after treatment †

Liver Function Test	Pre treatment Mean±SD/Range	Post treatment Mean±SD/Range	P-value
AST	31.1±20.3/ (10-156)	22.8±9.9/ (10-76)	P<0.0001
ALT	40.5±29.7/ (11-153)	26.8±15.7/ (10-95)	P<0.0001
Alk.P	187.2±63.5/ (33-361)	190.2±51.4/ (94-341)	P=0.615

* 100 patients with NAFLD

† Patients, who had carotid intima- media thickness in their color doppler sonography, received treatment

**Table 3:** Comparison of patients*’ carotid intima media thickness before and after treatment †

carotid intima media thickness(mm)	Pre treatment Mean±SD/Range	Post treatment Mean±SD/Range	P-value
Right Carotid Intima Media	0.55±0.12/(0.4-1.1)	0.52±0.11/(0.35-1.1)	P=0.001
Left Carotid Intima Media	0.57±0.12/(0.4-1.1)	0.53±0.09/(0.35-1)	P<0.0001

* 100 patients with NAFLD

† Patients, who had carotid intima- media thickness in their color Doppler sonography, received treatment

## Discussion

In this cross - sectional study, as a part of a 10-year cohort study, one year after drug therapy of fatty liver in patients with NAFLD, intima-media thickness of patients' carotids was significantly decreased in both left and right carotids. Similarly, the changes in patients' liver function tests were statistically meaningful. 

In the process of treating NAFLD, paying attention to the patients' weight loss, their blood sugar, metabolic syndrome and hyperlipidemia control as well as using antioxidants, such as vitamin E and drugs increasing insulin sensitivity have been recommended. To confirm the above sentences, before treatment, the mean of our patients' body mass index (BMI) was 29.9±4. So, our patients' overweightness revealed their need to being treated. 

In this study, vitamin E was prescribed at a dose of 400 mg and in various studies, in any age group and in no circumstances, no adverse effects have been reported with this dose. Indeed, a few rare cases of cerebro- vascular complications and sudden cardiac death have been reported with long-term administration of doses greater than 800 mg of vitamin E. 

To treat fatty liver in patients with NAFLD, drugs such as rosiglitazone and pioglitazone, which are thiazolidinon derivatives, have been proposed, too. However, their efficacy requires to be tested in some comprehensive reviews (7). Contrary to the above study, none of the patients took rosiglitazone and pioglitazone in our study. 

Early diagnosis of atherosclerosis process early in life can be helpful in prevention of its complications such as cardiovascular disease, hypertension, lipid disorders, liver disease, diabetes and cholestasis later in life (8). Since an increase in IMT in stages I and II shows atherosclerotic changes, it can be concluded that the incidence and severity of atherosclerotic changes of obesity begin in the adolescence (9). Targher and colleagues in 2007 showed that NAFLD was associated with increasing of IMT (as an early marker of generalized atherosclerosis). Therefore, it is inferred that most of the patients with NAFLD could be threatened by cardiovascular risks (8). According to the findings in Table 3, our patients' intima-media thickness before treatment was high as well and its decrease after drug therapy was meaningful. 

Generally, regarding the easy detection of fatty liver by ultrasound and its relationship with some components of metabolic syndrome, including abdominal obesity, high triglycerides and cholesterol, it is essential that any patient with a fatty liver on ultrasound should be examined in terms of carotid IMT and other criteria to the metabolic syndrome to prevent the cardiovascular risks.

In a study conducted by Salonen and associates in 2003, the effect of combined vitamin C and E supplementation on intima- media thickness and atherosclerotic progression was reviewed. Those who participated in their study had high cholesterol. In that study, it was found that the simultaneous intake of vitamins C and E might be able to slow progression of atherosclerosis. Moreover, patients’ intima- media thickness was significantly reduced after taking those vitamins (10). Similarly, in our study, the reduction of the patients’ carotid media thickness after one year treatment was statistically significant in both right and left carotids ( P<0/0001).

In a 2007 study, Crouse and colleagues studied the effect of Rosuvastatin on the progression of atherosclerosis. The results of their study showed that taking Rosuvastatin significantly reduced the rate of atherosclerosis progression. That study revealed that Carotid intima- media thickness was significantly reduced in the patients who have used Rosuvastatin (11). In contrast to the above study, none of the patients in our study took Rosuvastatin and patients were treated with some other various drugs such as, UDCA, vitamin E, vitamin C and metformin for one year.

In a study in 2010, the effect of L - carnitine supplementation was examined on the diet of patients with NAFLD. In that study, the reduction in CRP and TNF-α as well as other liver enzymes was measured after the treatment of non-alcoholic fatty liver disease. According to their findings, there were significant decrease in the above mentioned markers, considerable improvement of lipid profile and fatty liver histologic appearance in the patients in the intervention group, who received L – carnitine. The differences between intervention and control groups were statistically significant (p<0/0001) (12). In our study, without using L-carnitine and other treatments for fatty liver, the reduced levels of liver enzymes such as ALT and AST in patients was significant after one year (p<0/0001). Mean ALT in the patients with fatty liver was 40/5 ±29/7 before treatment and it was reached to 26/8 ± 15/7 mg dL one year after treatment. Mean AST in the patients was 31/1± 20/3 before treatment which was reached to 22/8± 9/9 mg dL.

In some studies, the combined treatment of fatty liver and its effects on carotid intima -media thickness have been examined. In a study conducted by Katakami and colleagues, the effect of metformin or glibenclamide and glitazone on slowing the progression of carotid intima- media thickness was compared. The results of that study showed that in patients who were taking metformin plus glibenclamide compared to those who were taking only glibenclamide, progression of carotid intima- media thickness was significantly reduced (13).

Since the aim of our study was not to assess the impact of such special treatment on carotid intima -media thickness, the impact of those medications on IMT was not examined separately. However, among the studied patients, 14 patients (14%) used combined (at least two) medications for the treatment of fatty liver. 

Sanei and associates conducted a similar study at Zahedan University of Medical Sciences in 2010. The results of their study indicated that in patients with nonalcoholic fatty liver, the values of common carotid intima- media thickness, waist circumference, waist-to-hip ratio (WHR), body mass index, triglycerides and total cholesterol were significantly higher than the control group. They suggested that the IMT of the carotid artery should be a part of the common review process in patients who suffer from NAFLD or metabolic syndrome (9). In contrary to our study, in their study, the impact of carotid intima- media thickness on the treatment of fatty liver was not examined and just values of IMT in patients with NAFLD was descriptively compared to those of control subjects. According to the findings, it can be concluded that treatment of fatty liver in patients with nonalcoholic fatty liver has a significant role in reduction of their carotid intima -media thickness and consequently in reduction cerebrovascular events such as stroke. Doing multi-center studies with a higher number of participants are recommended to confirm the findings of this study.
